# Changes of hepatic biochemical parameters and proteomics in broilers with cold-induced ascites

**DOI:** 10.1186/2049-1891-3-41

**Published:** 2012-12-11

**Authors:** Yongwei Wang, Yuming Guo, Dong Ning, Yunzhi Peng, Hong Cai, Jianzhuang Tan, Ying Yang, Dan Liu

**Affiliations:** 1 State Key Lab of Animal Nutrition, College of Animal Science and Technology, China Agricultural University, Beijing 100193, PR China

**Keywords:** Ascites, Broilers, Biochemical parameters, Proteomics analysis

## Abstract

Ascites syndrome is still a problem for chicken industry in various parts of the world. Despite the intensive investigations of this syndrome for many years, its pathogenesis remains unclear. The objective of this study was to analyze the difference in hepatic proteomics between ascites and healthy broilers by two-dimensional electrophoresis (2-DE) and matrix-assisted laser desorption/ionization-time of flight mass spectrometry (MALDI-TOF-MS). Changes of biochemical parameters of liver and blood were also determined. The results indicated that red blood cell counts (RBC), hematocrit (HCT) and haemoglobin (HGB) of ascites broilers were significantly greater than healthy broilers. Hepatic malondialdehyde (MDA) level of ascites broilers was significantly increased, and the activity of total superoxide dismutase (T-SOD) was significantly decreased. Hepatic lactic acid (LD) level of ascitic broilers were significantly lower than healthy ones. Serum glucose and cholesterol level of ascites broilers were significantly increased, and serum globulin level was significantly decreased in ascites broilers. There was no significant difference in triglyceride (TG) and blood urea nitrogen (BUN) level. The activity of liver hexokinase (HK) and succinodehydrogenase (SDH) in ascites broilers was significantly decreased, and there was no significant difference in the activity of liver pyruvate kinase (PK) and Na^+^-K^+^-ATPase. The hepatic proteomics analysis showed that 18 proteins expression difference were identified between ascites and healthy broilers. These proteins were mainly involved in: 1) cytoskeleton; 2) glucose, lipids and amino acid metabolism; 3) cell secretion; 4) cell apoptosis; 5) signal transduction; 6) immune and inflammatory response; and 7) cellular redox homeostasis. Mitochondrial isoform phosphoenolpyruvate carboxykinase (M-PEPCK) mainly participates in gluconeogenesis of chicken liver. In conclusion, liver oxidative damage was significantly aggravated, but antioxidant capacity was decreased in cold-induced ascites broilers. Serum glucose level was significantly increased, with liver M-PEPCK expression higher in ascites broilers, which implied that some potential regulatory reagents may reduce ascites susceptibility and mortality under cold temperature by increasing liver gluconeogenesis level.

## Introduction

It is reported that 5% of broilers and 20% of roaster birds die of ascites, the economic loss due to ascites was significant [1]. Despite intensive investigations of the syndrome for decades, the pathogenesis and underlying mechanisms of ascites is yet to be understood [2-6]. It has been shown that histologic lesions [7], significant reduction of hemoglobin content respective to red blood cell count [8,9], decreased production of vessel dilating active substances [10], abnormal metabolism of corticosteroids and triiodothyronine [9], and down-regulation of lung inflammatory chemokine genes [11], are involved in the occurrence of ascites.

The profound changes of liver glucose and lipids metabolism are associated with the metabolic abnormalities (such as obesity and diabetes), and differentially expressed proteins are responsible for the metabolic disorder [12,13]. Cisar et al. (2005) studied the differentially expressed proteins in myocardial mitochondria matrix between ascites-resistant and ascites-susceptible line broilers with and without ascites using 2-DE, the results indicated that mitochondria of ascites broilers may inappropriately respond to hypoxia [14]. However, hepatic differentially expressed proteins in ascites broilers were not yet identified.

Cold temperature can increase ascites susceptibility by increasing metabolic oxygen requirements [15]. However, there is little information about the changes of physiological parameters such as fasting blood glucose, serum protein, triglyceride, liver antioxidative capacity and metabolic enzymes activities between ascites and healthy broilers under a cold environment [16-18].

The objective of this study was to investigate the changes of hepatic proteomics profile, and changes of biochemical parameters of blood and liver tissue between ascites and healthy broilers under a low temperature environment.

## Materials and methods

### Experimental design

A total of 196 day-old male broilers (Ross-308) were randomly assigned to fourteen replicate cages (2.4 × 0.6 × 0.6 m) of 14 birds each. The temperature in the house was 35°C during the first week, and was lowered by 1°C every other day till 30°C was reached on day 10. From day 11 to the end of experiment (42 days), all birds were exposed to a temperature regime of 17°C during the day and 14°C at night in order to increase ascites susceptibility. Diets (Table 1) were formulated to meet or exceed recommended requirements for all nutrients [19] and were pelleted. Birds had free access to feed and water, with 23 h fluorescent illumination per day throughout the experimental period. Birds were vaccinated with newcastle disease-infectious bronchitis (ND-IB) vaccine at 7 and 21 days age, and infectious bursal disease (IBD) vaccine at 14 and 28 days age. The study protocol was approved and conducted in accordance with the Animal Ethics Committee guidelines of China Agricultural University.

**Table 1:** Composition of experimental diets

**Items**	**Day 1-21**	**Day 22-42**
Ingredient (%)		
Corn	50.44	59.40
Soybean meal, 45%	27.88	22.00
Corn gluten meal, 58%	12.00	11.03
Soybean oil	5.03	3.57
Dicalcium phosphate phosphate	1.89	1.42
Limestone	1.25	1.31
Salt	0.50	0.50
Mineral premix^a^	0.20	0.20
Vitamin premix^b^	0.03	0.02
Choline chloride (50%)	0.10	0.16
Aureomycin (15%)	0.10	0.10
Ethoxyquin (33%)	0.03	0.03
L-lysine	0.45	0.24
DL-Methionine	0.10	0.02
Total	100.00	100.00
Calculated analysis		
ME, Mcal/kg	3.20	3.20
CP (%)	23.00	20.00
Ca (%)	1.00	0.90
Available P (%)	0.45	0.35
Lysine (%)	1.25	1.00
Methionine (%)	0.50	0.38

^a^ supplied the following per kg complete diet: Cu, 8 mg; Zn, 75 mg; Fe, 80 mg; Mn, 100 mg; Se, 0.15 mg; I, 0.35 mg.

^b^ supplied the following (per kg complete diet): vitamin A, 12500 IU; vitamin D_3_, 2500 IU; vitamin E, 30 IU; vitamin K_3_, 2.65 mg; thiamine, 2 mg; riboflavin, 6 mg; vitamin B_12_, 0.025 mg; biotin, 0.0325 mg; folic acid, 1.25 mg; pantothenic acid, 12 mg; niacin, 50 mg.

### Sampling and measuement

#### Blood biochemical parameters and organ index

On days 39 (the age at which a peak in the syndrome was demonstrated previously [20], 80 birds were selected and weighted after 8 h of feed deprivation. Whole blood samples were collected by venipuncture into EDTA-K_3_ anticoagulation tubes for the measurement of RBC, HCT and HGB (Sysmex KX-21 N Automatic blood analyzer, Kobe, Japan). Another set of blood samples were immediately collected into non-anticoagulant tubes to obtain serum for the determination of glucose, total protein, globulin, triglyceride, cholesterol (Unicel DXC 800, Beckman Coulter, California, America). Blood urea nitrogen (BUN) was measured using commercially available colorimetric diagnostic kits as manufacturer’s instructions (Nanjing Jiancheng Bioengineering Institute, Nanjing, China).

Then the birds were killed by jugular bleeding. Liver, lung, and heart were separated to calculate liver index, lung index, and heart index, respectively. Weights of right ventricle (RV) and total ventricle (TV) were recorded to calculate the ascites heart index (AHI). AHI was calculated as (RV/TV) × 100 [21].

#### Hepatic biochemical parameters

According to the following selection criteria, liver tissue of eight ascites and healthy broilers were collected for the determination of MDA and LD level, and the activity of T-SOD, HK, SDH, PK and Na^+^-K^+^-ATPase. Liver tissues were minced and homogenized (10%, wt/vol) in physiological saline water at 4°C, then centrifuged at 3,500 g for 15 min at 4°C. The supernatant was collected for the determination of biochemical parameters. MDA level was determined with the method of thibabituric acid (cat^#^: A003-1). The activity of T-SOD (cat^#^: A001-1), HK (cat^#^: A077), SDH (cat^#^: A022), PK (cat^#^: A076) and Na^+^-K^+^-ATPase (cat^#^: A016-2), and the level of liver LD (cat^#^: A019-2) were measured using commercially available colorimetric diagnostic kits (Nanjing Jiancheng Bioengineering Institute, Nanjing, China) [22]. The protein content of liver tissue was measured by Coomassie Brilliant Blue G-250 reagent with bovine serum albumin as a standard.

The selection criteria was based on the following three aspects: (1) HCT < 0.36, healthy; HCT ≥ 0.36, ascites; (2) AHI < 0.28, healthy; AHI ≥ 0.28, ascites; (3) Having effusion in abdominal cavity and pericardium, ascites, no effusion, healthy.

#### Hepatic proteomic analysis

Another set of liver tissue were put in tubes, then snap frozen in liquid N_2_ and stored at −80°C. Based on the selection criteria mentioned above, liver tissue of four ascites and four healthy broilers were selected for the analysis of liver proteomics profile.

#### Protein sample preparation

Liver tissues were homogenized in a lysis buffer consisting of 40 mM Tris, 7 M Urea, 2 M Thiourea, 4% CHAPS, 1% Dithiothreitol (DTT), 1 mM EDTA-2Na, using a glass homogenization vessel in an ice bath. Tissues were ruptured using an ultrasonic cell disruptor at 0°C for 10 min with 2-s on and 8-s off cycles. Then the lysed cell suspension was kept at 4°C for 2 h to solubilize proteins. Subsequently, the homogenate was centrifuged at 4°C and 12,000 g for 5 min, finally the supernatant fluid was collected. Protein concentration for each of the final supernatants was determined by Bradford assay using bovine serum albumin as the standard. Protein extracts were stored in aliquots (1.5 mg/mL) at −80°C.

#### Two dimensional electrophoresis

This study consists of two groups with four replicates, so a total 8 gels were run for the 2-DE, using commercial IPG strips (pH 3-10 NL, 24 cm, GE Healthcare, Piscataway, NJ) for isoelectric focusing electrophoresis (IEF) and then standard vertical SDS-PAGE (13%) for second dimensional electrophoresis. Briefly, 1.5 mg protein sample was loaded onto IPG drystrips using the in-gel sample rehydration technique, according to manufacturer’s instructions. After rehydration for 12 h, the first-dimensional IEF was carried out at 20°C for 100,000 Vh in the Ettan IPGphorII IEF system (GE Healthcare, Piscataway, NJ) for 24 h. Sequentially, IPG strips were equilibrated for 15 min in 8 mL of equilibration buffer-1 (0.6 M urea, 2% DTT, 30% glycerol, and 50 mM Tris-Cl, 2% DTT, 0.07 M SDS, trace bromophenol blue, pH 8.8) and then in 8 mL of equilibration buffer-2 (0.6 M urea, 0.1 M iodoacetamide, 30% glycerol, and 50 mM Tris-Cl, 2% DTT, 0.07 M SDS, trace bromophenol blue, pH 8.8) for 15 min. The second dimensional electrophoresis was carried out on an Ettan DALTsix system (GE Healthcare) at 20°C for 7 h (4 w/gel). Then the gels were stained with colloidal Coomassie Brilliant Blue R-250 (Amresco, Inc., Solon, OH).

#### Image analysis

The gel images were obtained using an UMAX Imagescanner (Model Powerlook 2100XL, UMAX Technologies, Atlanta, America) and image analysis was performed using Imagemaster Platinum Version 7.0 software (GE Healthcare). After normalizing the quantity of each spot by total valid spot intensity, differentially expressed protein spot with the point value (the relative expression volume ratio) over 2.0-fold were selected and subjected to identification by mass spectrometry (MS).

#### In-gel digestion

Protein spots were manually obtained and destained with 100 μL destaining solution (acetonitrile (ACN):100 mM ammonium bicarbonate = 3:7) for 1 h, then dehydrated with 100 μL ACN for 15 min. The protein samples were completely dried by vacuum centrifugation for 5 min, and subsequently digested with 10 μL of Trypsin (Amresco, Inc., Solon, OH) in 100 mM ammonium bicarbonate at 4°C for 45 min, and incubated at 37°C for 16 h. The resulting peptides were subjected to sequential extraction with 70% ACN and 0.1% trifluoroacetic acid (TFA) for 30 min (3 times at 37°C), then the three retain solutions were collected together and dried until 5-6 μL left with a vacuum centrifugation (Labconco).

#### Protein identification by MS and database queries

Protein identification was carried out on a Matrix Assisted Laser Desorption Ionization-Time of Flight MS (MALDI-TOF-MS) (Autoflex II, Bruker, Germany). MS fingerprinting data were performed by search engine Mascot ( http://www.matrixscience.com), and Bony Vertebrates taxonomy against the NCBInr database. Search parameters include: (1) trypsin, as the enzyme of protein digestion; (2) MH^+^ and monoisotopic, as mass value; (3) unrestricted, as peptide mass; (4) ±0.3 Da, as peptide mass tolerance; (5) carbamidomethyl (C), as fixed modifications; oxidation (M), as variable modifications; and (6) 1, as maximum missed cleavages.

#### Statistical analysis

The biochemical parameters data were presented as Mean. The statistical analysis was performed with SPSS 16.0 software for Windows [23]. Independent sample *T*-test was used and differences were considered statistically significant at *P* ≤ 0.05.

## Results

### Differences of body weight and organ index between ascites and healthy broilers

As shown in Table 2, body weight of ascites broiler was significantly lower than healthy broilers, and liver index, heart index and AHI of ascites broilers were significantly increased, but there was no difference in lung index between ascites and healthy broilers.

**Table 2:** Changes of body weight and organ index between ascites and healthy broilers (n = 8)

**Items**	**BW (kg)**	**LI**	**LUI**	**HI**	**AHI**
Healthy	2.14^*^	2.62	0.24	0.49	22.2
Ascites	1.65	3.08^*^	0.25	0.60^*^	29.1^*^
SEM	0.09	0.09	0.01	0.02	1.45
p-value	0.003	0.006	NS	0.003	0.009

BW: Body weight, LI: Liver index, LUI: Lung index, HI: Heart index, AHI: Ascites heart index. ^*^P<0.05.

### Changes of blood biochemical parameters between ascites and healthy broilers

The data of blood biochemical parameters were shown in Table 3. RBC and HCT of ascites broilers were significantly greater than healthy broilers. Serum glucose, total cholesterol level of ascites broilers was significantly increased, and serum globulin level was significantly decreased, but there was no significant difference in triglyceride and BUN level.

**Table 3:** Changes of blood biochemical parameters between ascites and healthy broilers (n = 8)

**Items**	**RBC**	**HCT**	**HGB**	**GLU**	**TG**	**CHO**	**TP**	**GLO**	**BUN**
	**10**^**12**^**/L**		**g/L**	**mmol/L**	**mmol/L**	**mmol/L**	**g/L**	**g/L**	**mg/L**
Healthy	2.63	0.33	106	13.9	253	7.37	35.5	19.0^*^	4.81
Ascites	3.52^*^	0.44^*^	145	17.4^*^	275	10.3^*^	32.3	16.4	5.16
SEM	0.19	0.03	8.04	0.72	21.1	0.52	1.53	0.87	0.38
p-value	0.001	0.002	0.021	0.008	NS	0.001	NS	0.016	NS

RBC: Red blood cells, HCT: Hematocrit, HGB: Haemoglobin,GLU: Glucose,TG: Triglyceride,CHO: Cholesterol, TP: Total protein, GLO:Globulin, BUN: Blood urea nitrogen.

### Changes of liver biochemical parameters between ascites and healthy broilers

Liver MDA level of ascites broilers was significantly increased, and T-SOD activity was significantly lower than in healthy broilers. The activity of liver HK, SDH in ascites broilers was significantly decreased, and there was no significant difference in the activity of liver PK and Na^+^-K^+^-ATPase. Liver LD level of ascitic broilers were significantly lower than healthy ones (Table 4).

**Table 4:** Changes of liver biochemical parameters between ascites and healthy broilers (n = 8)

**Items**	**MDA**	**T-SOD**	**HK**	**SDH**	**PK**	**Na**^**+**^**-K**^**+**^**-ATPase**	**LD**
	**nmol/mg protein**	**U/mg protein**	**U/mg protein**	**U/mg protein**	**U/g protein**	**μmolPi/mg protein/h**	**mmol/mg protein**
Healthy	0.27	4.63^*^	65.14^*^	5.53^*^	34.9	6.29	0.20
Ascites	0.31^*^	3.74	41.35	3.99	29.8	8.01	0.16^*^
SEM	0.01	0.18	4.70	0.38	1.87	0.89	0.012
p-value	0.012	0.005	0.01	0.038	NS	NS	0.044

MDA: Malondialdehyde, T-SOD: Total superoxide dismutase, HK: Hexokinase, SDH: Succinodehydrogenase, MDH: Malic dehydrogenase, PK: Pyruvate kinase, LD: Lactic acid.

### Changes of liver proteomics profiles between ascites and healthy broilers

**Figure 1:**
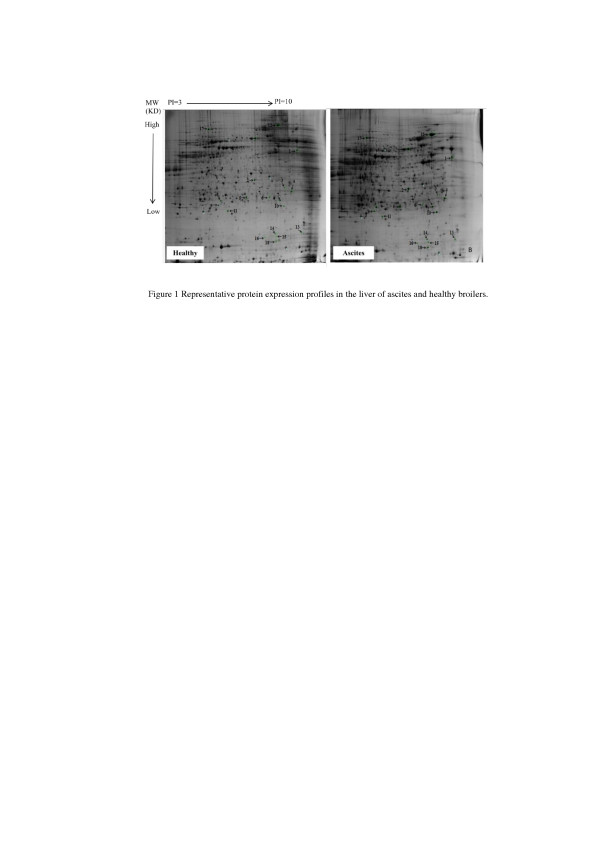
Representative protein expression profiles in the liver of ascites and healthy broilers.

**Figure 2:**
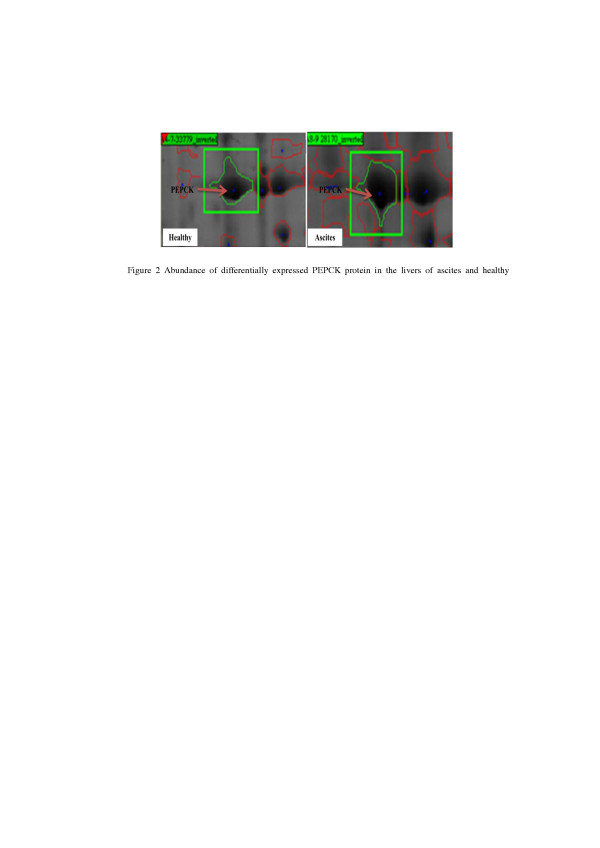
Abundance of differentially expressed PEPCK protein in the livers of ascites and healthy broilers.

**Figure 3:**
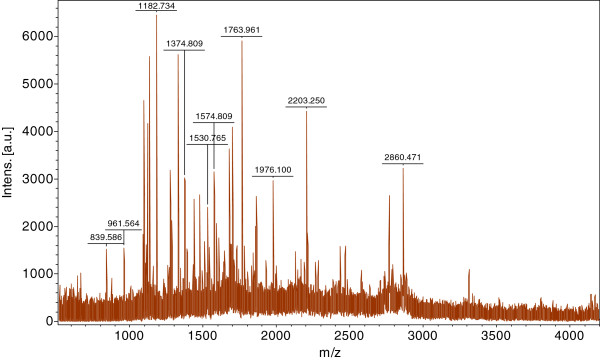
MALDI-TOF mass spectrum of M-PEPCK from a 2D gel.

The results of liver proteomics analysis indicated that 18 proteins expression difference were identified between ascites and healthy broilers (Figure 1), including keratin, annexin, chicken M-PEPCK chain A, dihydropteridine reductase (DHPR), N-ethylmaleimide-sensitive factor (NSF), translationally-controlled tumor protein homolog (TCTP), ring finger protein 170, Rho GDP dissociation inhibitor alpha (GDI-α), ryncolin-1, natural killer cell enhancing factor isoform 4 (NKEF), glia maturation factor beta (GMF), neutrophil cytosol factor 2 variant (NCF) and two unnamed proteins (Table 5). Chicken M-PEPCK was mainly involved in liver gluconeogenesis (Figure 2 and Figure 3). The result of the current study revealed that the expression of liver M-PEPCK chain A in ascites broilers was higher than in healthy broilers (Table 6).

**Table 5:** Liver differentially expressed proteins of broilers identified by PMF query

**Match ID**	**Score**	**Name**	**Id**	**Mass**	**Pi**	**Coverage (%)**
882	89	similar to keratin 10 isoform 2	gi|21961605	56863	5.05	22
520	98	chain A, crystal structures of chicken annexinV in complex with Ca^2+^	gi|62738641	36159	5.61	39
443	91	similar to proteasome subunit alpha type 3	gi|73963044	15430	7.77	53
409	72	keratin, type II cytoskeletal 1	gi|160961491	65621	7.62	20
333	55	N-ethylmaleimide-sensitive factor	gi|118102799	82638	6.38	18
311	57	ring finger protein 170, isoform CRA_b	gi|119583606	22591	8.6	28
304	75	Rho GDP dissociation inhibitoralpha	gi|224074434	23247	5.02	34
294	117	dihydropteridinereductase	gi|57529509	25107	6.43	34
252	53	ryncolin-1	gi|299791444	38896	5.42	22
248	97	translationally-controlled tumor protein homolog	gi|45382329	19689	4.9	25
213	110	similar to natural killer cell enhancing factor isoform 4	gi|50751518	22529	8.24	53
1895	264	unnamed protein product	gi|12835845	56972	5.01	37
144	60	neutrophil cytosol factor 2 variant	gi|62088874	68173	5.9	22
136	60	glia maturation factor beta	gi|71894963	16884	5.19	54
130	118	keratin, type II cytoskeletal 1	gi|160961491	65621	7.62	28
125	49	serpin B3	gi|41235787	44800	8.58	25
1093	302	chain a, the structure of chicken mitochondrial PEPCK.	gi|110591367	68010	6.55	48
108	52	hypothetical protein LOC100446659	gi|297668385	23305	9.12	23

**Table 6:** Expression volume of liver different protein spots in 2 DE map

**Spot No.**	**Protein name**	Expression volume^*^		***p*-value**
		**Healthy**	**Ascites**	
1	similar to keratin 10 isoform 2	0.141	0.150	0.081
2	chain A, crystal structures of chicken annexin v in complex with Ca^2+^	0.042	0.057	0.076
3	similar to proteasome subunit alpha type 3	0.053	0.063	0.120
4	keratin, type II cytoskeletal 1	0.085	0.077	0.041
5	N-ethylmaleimide-sensitive factor	0.051	0.071	0.045
6	ring finger protein 170, isoform CRA_b	0.088	0.049	0.004
7	Rho GDP dissociation inhibitor (GDI) alpha	0.120	0.156	0.002
8	dihydropteridinereductase	0.122	0.128	0.053
9	ryncolin-1	0.080	0.121	0.004
10	translationally-controlled tumor protein homolog	0.131	0.160	0.014
11	similar to natural killer cell enhancing factor isoform 4	0.126	0.074	0.027
12	unnamed protein product	0.214	0.228	0.109
13	neutrophil cytosol factor 2 variant	0.129	0.150	0.216
14	glia maturation factor beta	0.046	0.046	0.039
15	keratin, type II cytoskeletal 1	0.193	0.387	0.177
16	serpin B3	0.171	0.231	0.057
17	chain A, the structure of chicken mitochondrial PEPCK.	0.084	0.130	0.006
18	hypothetical protein LOC100446659	0.432	0.894	0.114

^*^means the volume of protein spot in 2D gel calculated by imagemaster Platinum Version 7.0 software.

## Discussion

### Organs index and blood parameters

The ascites syndrome in broilers is attributed to higher metabolic burdening, so one of the pathogenesis of the syndrome is metabolic hypoxia or low efficiency in oxygen utilization [2]. In the case of hypoxia, broilers have difficulties in fulfilling tissue demands for oxygen, and the birds exhibit the decreased blood saturation and increased production of RBC (partially immature) with decreased hemoglobin content relative to red blood cells, so the decrease in hemoglobin might have contributed to enhanced development of hypoxemia and aggravation of ascites [9,24]. Luger et al. (2003) found RBC and HCT were significantly increased, and with no significant change in plasma volume in ascites chickens at wks 5, which was consistent with the current study. Besides, continually increased corticosterone level might be an inducer of erythropoiesis proliferation and differentiation arrest in ascites broilers [9]. Although increased haematrocrit values (and presumably increased haemoglobin) should be positive to alleviate hypoxaemia, birds dying of ascites all showed high haematocrits, which increased blood viscosity and augmented the inability of the failing right ventricle to pump blood through vasconstricted pulmonary blood vessels [8,25]. The liver and heart are the important organs to consume oxygen. Many studies take AHI > 0.27-0.30 as an indicator of ascites occurrence, with right ventricular hypertrophy and pulmonary hypertension [26]. Hypertrophy of organs was a sign of abnormal function leading to the decrease in growth performance [7]. Results of the present study indicated that liver and heart of ascites broilers was significantly hypertrophic with the lower body weight, which increased the susceptibility to ascites and aggravated the occurrence of ascites syndrome.

### Lipid antioxidant capacity and enzymes activity related to energy metabolism

Oxidative stress was aggravated in ascites broilers [27]. Ascites syndrome induced a large number of reactive oxygen species (ROS) and MDA production in many tissues, causing lipid peroxidation in mitochondrial membrane, leading to over-consumption of antioxidant enzymes and inadequately synthesize. The concentration of free radical and MDA were increased in serum and other tissues, but the activity of SOD and GSH-Px was significantly reduced [28]. The current study indicated that hepatic MDA concentration of ascites broilers was increased, and T-SOD activity was significantly decreased, which indicated that hepatic damage due to lipid peroxidation was significantly aggravated in ascites broilers. The scavenging agents of free radical, such as Vitamin E [29,30], or L-carnitine can enhance the defense function of antioxidative system, and reduce the susceptibility to ascites [28].

Cold temperature can increase ascites susceptibility by increasing both metabolic oxygen requirements and pulmonary hypertension [15]. HK, SDH and PK are the important enzymes in cell glycolysis and Krebs cycle. The activity of these enzymes is of importance to glucose catabolism. Na^+^-K^+^-ATPase activity is an important indicator to measure mitochondria function for energy metabolism, and it has close relationship with the decomposition of ATP. The current study indicated that the activity of liver mitochondria enzymes was significantly decreased, which might block the ATP generation process in liver tissue of ascites broilers, so the energy requirement cannot be fulfilled under a cold environment, which may aggravate the development of ascites.

### Differentially expressed proteins indentified from proteomics profile

18 proteins expression difference were identified between ascites and healthy broilers. These proteins were mainly associated with the following biological processes: 1) cytoskeleton; 2) glucose, lipids and amino acid metabolism; 3) cell secretion; 4) cell apoptosis; 5) signal transduction; 6) immune and inflammatory response; and 7) cellular redox homeostasis. However, there are few reports on the relationship between the functions of differentially expressed proteins and ascites syndrome. It is reported that PEPCK, rather than being involved exclusively in gluconeogenesis, has a broader metabolic function in the cataplerosis of citric acid cycle intermediates (removal of citric acid cycle anions), which is required for gluconeogenesis and glyceroneogenesis [31]. So we are very interested in the PEPCK.

It is almost 60 years since the M-PEPCK (EC 4.1.1.32) from chicken liver was first reported by Utter and Kurahashi [32]. Most mammalian species display almost equal activity of the two isoforms of PEPCK in their liver, one in mitochondria (M-PEPCK) and another in cytosol (C-PEPCK), whereas chicken only express the gene for M-PEPCK in their liver (The expression of liver C-PEPCK decreases to negligible levels 2 d before hatching) [33-35]. In chicken, glucose synthesis in the liver is restricted to the Cori cycle, whereas net gluconeogenesis from amino acids occurs in the kidney, a tissue that contains both isoforms of PEPCK. Thus, birds recycle lactate generated by glycolysis in the muscle and red cells to the liver for gluconeogenesis [33,36]. These differences in the regulation of gluconeogenesis may be related to the different ways of generating cytosolic NADH when specific metabolic precursors are used in gluconeogenesis [37]. In avian liver, the synthesis of phosphoenolpyruvate is restricted to the mitochondria, so that a continuous supply of NADH from oxidation-reduction reactions in the cytosol is needed. In tissues that possess a C-PEPCK, malate is transported across the mitochondrial membrane, generating NADH in the cytosol where it is oxidized to oxaloacetate, then a variety of gluconeogenic precursors can be used (alanine, pyruvate and gluconeogenic amino acids) [34].

Daneshyar et al. (2009) reported that fasting blood sugar of cold-temperature treated birds at wks 4 and wks 6 was significantly greater than normal-temperature treated birds, and they attributed it to higher gluconeogenesis level in ascites broilers [16,38]. Besides, Yersin et al. (1992) and Biswas et al. (1995) observed decreased concentration of serum albumin in ascites broilers. It is suggested that deamidation process might contribute to the high rate of gluconeogenesis in hepatocytes of ascites broilers [17,18]. This study found that serum globulin level was significantly decreased in ascites broilers. In addition, the expression of liver M-PEPCK in ascites broilers was significantly up-regulated, with the significant decrease of liver lactic acid level and increase of serum glucose level in ascites broilers. It is implied that the gluconeogenesis level was increased during the development of broilers ascites under a low ambient temperature.

In summary, the study provides the first evidence for the changes of hepatic proteomics profile between ascites and healthy broilers. Eighteen differentially expressed protein spots were identified between ascites and healthy broilers. It can be concluded that liver oxidation damage and energy generation obstruction occurred in ascites broilers. Hepatic M-PEPCK expression of ascites broilers was significantly increased-indicating that gluconeogenesis level was increased in ascites broilers under low ambient temperature. It is implied that some potential regulatory reagents may reduce ascites susceptibility by increasing gluconeogenesis level through up-regulating M-PEPCK protein expression.

## Endnotes

Data in this paper were partially presented at the 2012 Poultry Science Association Annual Meeting, Athens, Georgia, America (p149).

## Competing interests

The authors declare that they have no competing interests.

## Authors’ contribution

All authors read and approved the final manuscript.
